# Tetrodotoxin (TTX) as a Therapeutic Agent for Pain

**DOI:** 10.3390/md10020281

**Published:** 2012-01-31

**Authors:** Francisco Rafael Nieto, Enrique José Cobos, Miguel Ángel Tejada, Cristina Sánchez-Fernández, Rafael González-Cano, Cruz Miguel Cendán

**Affiliations:** Department of Pharmacology and Institute of Neuroscience, Faculty of Medicine, University of Granada (Avenida de Madrid 11, 18012 Granada, Spain) and Biomedical Research Center, University of Granada, Parque Tecnológico de Ciencias de la Salud, 18100 Armilla, Granada, Spain; Email: ejcobos@ugr.es (E.J.C.); mtejada@ugr.es (M.Á.T); csanchezf@ugr.es (C.S.-F.); rgcano@ugr.es (R.G.-C.)

**Keywords:** tetrodotoxin, TTX, TTX-sensitive voltage-gated sodium channels, pain, neuropathic pain

## Abstract

Tetrodotoxin (TTX) is a potent neurotoxin that blocks voltage-gated sodium channels (VGSCs). VGSCs play a critical role in neuronal function under both physiological and pathological conditions. TTX has been extensively used to functionally characterize VGSCs, which can be classified as TTX-sensitive or TTX-resistant channels according to their sensitivity to this toxin. Alterations in the expression and/or function of some specific TTX-sensitive VGSCs have been implicated in a number of chronic pain conditions. The administration of TTX at doses below those that interfere with the generation and conduction of action potentials in normal (non-injured) nerves has been used in humans and experimental animals under different pain conditions. These data indicate a role for TTX as a potential therapeutic agent for pain. This review focuses on the preclinical and clinical evidence supporting a potential analgesic role for TTX. In addition, the contribution of specific TTX-sensitive VGSCs to pain is reviewed.

## 1. Introduction

Pain is a perception and, as a part of the sensory system, has the important protective function of warning us from harm that should be avoided or treated, and hence prolong survival. In this context, behaviors resulting from pain facilitate fundamental biological actions such as the healing of damaged tissues. This kind of pain is essential for maintaining bodily integrity and is associated with noxious stimuli, and is therefore called nociceptive pain [1]. In general, nociceptive pain is not a clinical problem and properly resolves after the healing process has ended. Unfortunately, under certain conditions pain loses its protective role and becomes not only purposeless but also highly distressing. This pain condition is related to neuropathic pain, which describes pain occurring with an abnormally functioning somatosensory nervous system to contrast with the normal function seen in nociceptive pain [1].

Pain is an enormous global health problem. It has been estimated that 20% of adults suffer from pain globally and 10% are newly diagnosed with chronic pain each year [2]. In particular, chronic and neuropathic pain affects millions of people worldwide including causes such as cancer, osteo- and rheumatoid arthritis, operations and injuries, and spinal problems [2]. Therefore, there is an obvious need to identify and develop new analgesics in order to better treat these unrelieved pain conditions.

Tetrodotoxin (TTX) is a neurotoxin found in puffer fish and other marine and terrestrial animals and it has been extensively used to elucidate the role of specific voltage-gated sodium channels (VGSCs) subtypes in a wide range of physiological and pathophysiological processes in the nervous system [3]. VGSCs play a key role in pain and TTX-sensitive subtypes have received much attention over the past few years because these channels have been strongly implicated in normal and pathological pain [4]. Since TTX blocks this subset of VGSCs in a highly selective manner, this agent may have a potential role in relieving pain. In this review, we will examine the roles of TTX-sensitive VGSCs in pain and subsequently, we will highlight the evidences obtained in preclinical animal studies and those studies performed in humans supporting TTX as a potential analgesic compound.

## 2. Voltaged-Gated Sodium Channels and TTX

VGSCs are members of the ion channel protein superfamily and play an essential role in neuronal and non-neuronal function, being responsible for the initiation and propagation of action potentials in excitable cells by allowing the influx of sodium ions. The VGSCs are large integral membrane proteins composed of a 260 kDa α-subunit and one or more auxiliary β-subunits. The α-subunit is sufficient for functional expression, forming the pore, determining the biophysical properties of the channel, and containing the ion selectivity filter [5,6]. β-Subunits can modify the kinetic and voltage dependence properties of the channel and are involved in channel localization and interaction with cell adhesion molecules, extracellular matrix, and intracellular cytoskeleton [7]. 

**Figure 1:**
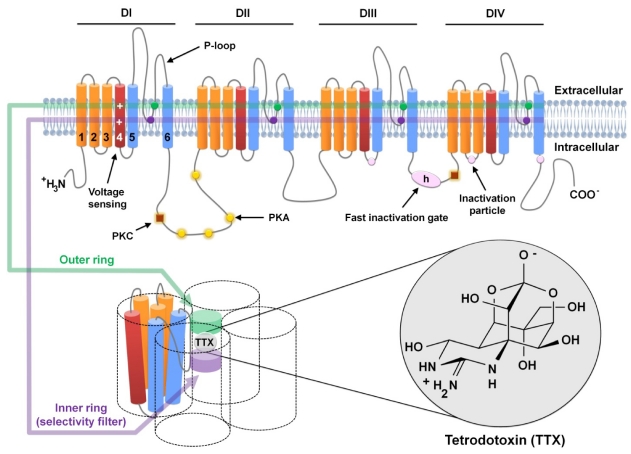
Schematic representation of voltage-gated sodium channel α-subunits and Tetrodotoxin (TTX) binding site. Voltage-gated sodium channel α-subunits are formed by four homologous domains (DI-IV), each consisting of 6 α-helical transmembrane segments (1–6). Segment 4 (dark red) corresponds to the voltage sensors. Sites of phosphorylation by protein kinase A (PKA) and protein kinase C (PKC) are represented by yellow circles and brown squares, respectively. The fast inactivation gate (IFM motif) is located in the intracellular loop between domains 3 and 4 and is represented by h (in pink oval); pink circles show the sites involved in forming the inactivation gate receptor. P-loops are located between helices 5 and 6 (in blue), which are the pore-lining segments (as shown in the lower figure). Outer (EEDD motif) and inner (DEKA motif) rings, represented by a green and purple band, respectively (in both the upper and lower figures), are formed by the amino acids indicated by circles of the same color. The TTX molecule interacts with the amino acid residues of these two rings in the pore of the channel, as detailed in the lower figure.

Nine mammalian α-subunit isoforms have been identified and functionally expressed, encoded by different genes that give rise to nine VGSC subtypes (Na_v_1.1–Na_v_1.9). A tenth isoform (Na_X_) has been recognized as a related protein that does not encode a VGSC [8]. Figure 1 depicts a schematic representation of α-subunits, which are large polypeptides that all share the following overall structure in common: four homologous domains (DI-DIV), each containing six transmembrane α-helical segments (S1–S6), which are connected by extracellular and intracellular loops. Specific amino acid sequences of the α-subunit form the pore wall of the ion channel, the voltage sensor, the inactivation gate, and the protein phosphorylation sites [9]. The α-subunit also contains the binding site for local anesthetic, anti-arrhythmic, and anti-epileptic drugs [10] and for several groups of neurotoxins that can markedly alter channel functions [11]. For a comprehensive review of VGSCs, the reader is referred to specific review articles [5,6,8,9].

TTX binds to neurotoxin receptor site 1 on the α-subunit within the outer vestibule of the VGSC and blocks the influx of sodium ions by occluding the outer pore of the channel [12]. This binding inhibits the propagation of action potentials, thereby paralyzing nerve and muscle function [13]. The nine mammalian VGSC subtypes that have been identified have distinct kinetics and voltage-dependent properties and differ in their tissue localization and sensitivity to TTX [8]. Nanomolar concentrations of TTX block Na_v_1.1, Na_v_1.2, Na_v_1.3, Na_v_1.4, Na_v_1.6, and Na_v_1.7 subtypes (TTX-sensitive VGSCs), whereas significantly higher (micromolar) concentrations are needed to block Na_v_1.5, Na_v_1.8 and Na_v_1.9 subtypes (TTX-resistant VGSCs) [8]. Therefore, in mammals, the physiological effects of TTX differ among various excitable tissues depending on the VGSC isoforms expressed in their cells [14]. 

The functional roles of VGSCs expressed by neurons are well-established (the generation and transmission of action potentials). VGSCs are also present in many non-neuronal cells within the nervous system and outside the nervous system, although their contributions to cellular functions of these cells are not fully understood [15,16]. In particular, TTX-sensitive (as well as TTX-resistant) VGSCs are expressed in some cell types within the central nervous system, such as astrocytes [17]. These kind of glial cells are not classically considered excitable, although they express VGSCs at levels that could support generation of action potential-like responses if resting inactivation is removed [18]. In addition, VGSCs appear to play significant roles in the function and viability of these cells [19]. Thus, clinical studies in which TTX or TTX-like agents are introduced into the central nervous system should carefully monitor for changes in neurological function.

VGSCs have received considerable attention for their therapeutic potential. Mutations in the genes encoding VGSCs (called “channelopathies”) have been identified as the cause of numerous hereditary diseases in heart, skeletal muscle, brain and peripheral nerves [20,21,22,23]. In addition, changes in the expression of non-mutated VGSC genes have been implicated in a number of disorders, including pain and multiple sclerosis [24]. In this review, we focus on VGSC research contributing evidence on the potential analgesic role of TTX.

## 3. TTX-Sensitive Voltage-Gated Sodium Channels and Pain

VGSCs play a key role in nociception, being one of the primary classes of ion channels responsible for driving noxious information to the central nervous system. Dysfunctional VGSCs have been related to several pain states, and data from human genetic studies and transgenic mouse models point to the implication of specific VGSC isoforms in particular types of pain [4,25]. The development of drugs that selectively block a single channel or selected channels is therefore of therapeutic interest and could reduce the adverse effects associated with non-selective sodium channel blockade [26,27].

As noted above, TTX is a highly selective blocker of a subset of VGSCs. Some of these TTX-sensitive VGSCs (e.g., Na_v_1.7) are preferentially expressed in adult sensory neurons and have been implicated in normal and pathological pain. It has also been reported that nerve injury is followed by upregulation of the expression of TTX-sensitive VGSCs (especially Na_v_1.3) in parallel with an increase in TTX-sensitive VGSC currents and a downregulation of TTX-resistant VGSC expression and currents [28,29,30,31,32]. This upregulation of TTX-sensitive VGSCs in adult sensory neurons leads to electrophysiological changes that may contribute to neuropathic pain states [33,34,35]. In particular, re-expression of the Na_v_1.3 α-subunit has been linked to the hyperexcitability and ectopic firing observed in spinal sensory neurons after injury [36]. Given the therapeutic potential of a drug that selectively blocks these VGSC subtypes, the possible role of TTX in pain treatment has been investigated in humans and animals, as is discussed in detail below (see Section 4 and Table 2). TTX-resistant VGSCs have also been proposed to participate in several pain states [4,25], but this review only reports evidence on the contribution of the TTX-sensitive subset of VGSCs. 

The TTX-sensitive VGSC subtype Na_v_1.4 is almost exclusively expressed in skeletal muscle [37], making its involvement in pain states highly unlikely. The other TTX-sensitive VGSCs (Na_v_1.1, Na_v_1.2, Na_v_1.3, Na_v_1.6 and Na_v_1.7) are well distributed throughout the central and/or peripheral nervous system (see Table 1), and their expression in neurons of the adult dorsal root ganglion (DRG) is especially well documented [38]. The contributions of these ion channels to different types of pain have been studied to a varying extent, as summarized in Table 1 and reviewed below.

**Table 1:** Summary of the potential implication of TTX-sensitive voltage-gated sodium channels in pain states.

Channel	Normal localization	Changes of expression in pain states	Knockdown/Knockout	Mutations related to pain states
**Na_v_1.1**	-CNS, PNS (in DRG mainly in A-fiber neurons) -Microglia	*Animal* -Unclear after PNI in NP	―	-Inherited hemiplegic migraine
**Na_v_1.2**	-Mainly CNS, very low expression in PNS -In SC in lamina I/II	*Animal* -Unclear after PNI in NP	―	―
**Na_v_1.3**	-Embryonic sodium channel -In adult neurons, in lamina I/II of SC, negligible in DRG	*Human* -↑ in human neuromas -↑ in human nerves after PNI -↑ in human trigeminal neuralgia *Animal* -↑ in DRG in inflammatory pain -↑ in DRG after PNI in NP -↑ in trigeminal ganglion after PNI in NP -↑ in SC dorsal horn after PNI in NP -↑ in rat neuromas -↓ in ferret trigeminal neuralgia	-Contradictory data with i.t. antisense ODNs -Knockout mice developed normally acute, inflammatory and neuropathic pain	―
**Na_v_1.4**	- In skeletal muscle	―	―	―
**Na_v_1.6**	-Mainly in Nodes of Ranvier -In SC and PNS (in DRG mainly in A-fiber neurons) -In epidermal free nerve terminals and keratinocytes -Main sodium channel in microglia	*Human* -↑ in skin of patients with complex regional pain syndrome and post-herpetic neuralgia *Animal* -Unclear in diabetic neuropathy -↑ in nerve after infraorbital nerve injury -↓ in DRG after PNI in NP	―	―
**Na_v_1.7**	-Mainly in PNS in all types of DRG neurons -In SC and PNS (in DRG, mainly in A-fiber neurons) -In epidermal free nerve terminals	*Human* -↑ in human neuromas -↑ in skin of patients with complex regional pain syndrome and post-herpetic neuralgia -↑ painful human dental pulp -↑ in idiopathic rectal hypersensitivity and fecal urgency -↓ in human DRG after PNI -↓ in human trigeminal neuralgia *Animal* -↑ in DRG in inflammatory pain -↑ in rat neuromas -Unclear in diabetic neuropathy -↓ in DRG after PNI in NP -↓ in sciatic nerve after PNI in NP -↓ in ferret trigeminal neuralgia	-Knockdown of Na_v_1.7 ↓ inflammatory pain and Na_v_1.7 expression in primary afferents in mice -Knockout mice showed ↑ mechanical and thermal pain thresholds and ↓ inflammatory pain - Knockout mice developed normally neuropathic pain	-Inherited erythermalgia -Paroxysmal extreme pain disorder -Congenital insensitivity to pain

CNS: central nervous system; DRG: dorsal root ganglia; i.t.: intrathecal; NP: neuropathic pain; ODN: Oligodeoxynucleotide; PNI: peripheral nerve injury; PNS: peripheral nervous system; SC: spinal cord.

### 3.1. Na_v_1.1

VGSC isoform Na_v_1.1 is extensively expressed in both the central and peripheral nervous system [8]. In the DRG, this sodium channel is expressed predominantly in large myelinated A-fiber neurons and only weakly in nociceptive neurons [39]. Na_v_1.1 is one of the TTX-sensitive sodium channel isoforms present in microglia [16], which play a role in pathologic pain [40]. Some mutations of the gene encoding for this channel (SCN1A) have been related to a type of inherited hemiplegic migraine [41]. Na_v_1.1 protein expression in neuroma tissue from patients with well-documented pain was similar to that of control patients [42], and Na_v_1.1 protein and mRNA expression in DRG neurons was unchanged in an animal model of carrageenan-induced inflammatory pain [43]. However, experimental models of neuropathic pain have yielded contradictory results. Thus, peripheral nerve injury was associated with no change in Na_v_1.1 expression of the ventral posterolateral nucleus of the thalamus [44], and with a decrease [32,45] in Na_v_1.1 mRNA expression or an increase [46] in its protein expression at DRG level. Further research is therefore required to clarify the role of this subunit in pathologic pain.

### 3.2. Na_v_1.2

Isoform Na_v_1.2 is one of the main VGSCs in the central nervous system [47]. The expression of its mRNA was unchanged in neurons of the ventral posterolateral nucleus of the thalamus after peripheral nerve injury in rat [44]. In DRG neurons, Na_v_1.2 expression was unchanged in a model of inflammatory pain [43] and either unaffected [45] or decreased [32] after peripheral nerve injury. No change in its expression was observed in painful human neuromas [42]. In addition, studies of the peripheral nervous system have generally reported very low signals of Na_v_1.2 at this level [32,38,39,42,43,45]. Hence, this subtype appears not to be involved in pain, and it has received little research attention in this setting. Nevertheless, Na_v_1.2 was very recently identified as one of the sodium channel isoforms that mediate action potential firing in lamina I/II spinal cord neurons, which are primarily composed of Na_v_1.2 and Na_v_1.3 isoforms [48].

### 3.3. Na_v_1.3

Isoform Na_v_1.3 is the main sodium channel in embryonic neurons [8] and is also expressed in the adult central nervous system, especially in humans [47]. In the adult rat, its expression is particularly concentrated in neurons of the superficial layers of the spinal cord dorsal horn [49,50], and the sodium channel currents of neurons in these layers (lamina I/II) are largely mediated by Na_v_1.3 along with Na_v_1.2 [48]. Based on this evidence of the functional expression of Na_v_1.3 in lamina I/II neurons of naïve rats, the latter group suggested that this isoform may play important roles in both acute and chronic pain signaling mechanisms.

As noted above, Na_v_1.3 is an embryonic sodium channel. However, its reexpression after nervous system injury has been demonstrated in numerous reports. Thus, authors have reported Na_v_1.3 upregulation (re-expression) in nerves from patients with peripheral axotomy *versus* control nerves [51], increased Na_v_1.3 mRNA expression in the gingival tissue of patients with trigeminal neuralgia [52], and increased Na_v_1.3 protein expression in painful human neuromas [42]. Na_v_1.3 expression was upregulated in DRG neurons in a rodent model of inflammatory pain [43], and in DRG sensory neurons [28,29,31,32,35,39,45,53,54,55,56,57,58,59,60,61,62,63,64], trigeminal ganglion [65], spinal cord dorsal horn [66,67], and thalamic nucleus [44,68], in a large number of experimental neuropathic pain models. In most of these studies, Na_v_1.3 upregulation was paralleled by an increase in pain behavior and/or electrophysiological changes, such as neuronal hyperresponsiveness or spontaneous firing activity. Interestingly, a rat study of several thousand of selected genes in the cell bodies of DRG sensory neurons after peripheral axotomy found changes in the expression of only 122 genes, including a 2-5 fold increase in expression of the gene for Na_v_1.3 and a decrease in expression of the gene for the TTX-resistant VGSC Na_v_1.8 [69].

Na_v_1.3 has several biophysical properties that contribute to neuronal hyperresponsiveness, and its increased expression in sensory/nociceptive neurons under pain conditions may be functionally significant [34,70]. In fact, Na_v_1.3 upregulation has been linked to an increase in persistent fast-activating and fast-inactivating TTX-sensitive sodium currents in DRG and spinal dorsal horn neurons, which likely contributes to the neuronal hyperresponsiveness responsible for allodynia and hyperalgesia after nerve injury [34,71].

Taken together, these findings suggest that re-expression of Na_v_1.3 in first-, second-, and third-order neurons along the pain axis might be involved in pathologic pain. However, contradictory reports have been published by other authors. For example, although the immunoreactive expression of Na_v_1.3 was found to be upregulated in neuromas from humans [42] and rats [35], little change was observed in neuromas from mice [72]. Moreover, rat studies found no significant change in Na_v_1.3 mRNA levels after unilateral sciatic nerve entrapment injury [73] or gradual elongation of sciatic nerve [74]. Downregulation of Na_v_1.3 was even reported in the trigeminal ganglia in a ferret model of trigeminal neuropathic pain [75]. The intrathecal administration of antisense oligodeoxynucleotides targeting Na_v_1.3 was reported to decrease Na_v_1.3 mRNA and protein expression, reducing hyperexcitability of dorsal horn neurons and attenuating neuropathic pain behavior after sciatic nerve and spinal cord injury [66,67]. However, other authors found no improvement in peripheral nerve injury-induced neuropathic pain after intrathecal administration of different antisense oligodeoxynucleotides selective for Na_v_1.3 [59]. In addition, a study in Dr. Wood’s laboratory [76] reported the normal development of acute and inflammatory pain in global Na_v_1.3 knockout mice and, more surprisingly, no modification of nerve injury-induced neuropathic pain behavior in global and nociceptive-specific Na_v_1.3 knockout mice. Therefore, despite the considerable evidence of an upregulation of Na_v_1.3 along the pain axis, it is likely that other VGSCs can also contribute to pathologic pain.

### 3.4. Na_v_1.6

Isoform Na_v_1.6 is mainly localized in nodes of Ranvier in the peripheral and central nervous system [77,78] and along non-myelinated axons [79], suggesting the importance of this sodium channel in nerve conduction. It is also well distributed throughout the spinal cord [50]. Its expression in the DRG is predominantly in large myelinated A-fiber neurons [39], as in the case of Na_v_1.1. Expression of Na_v_1.6 (and Na_v_1.7, Na_v_1.8, and Na_v_1.9) has been reported in axons composing small nerve bundles underlying the epidermis and in epidermal free nerve terminals, which include nociceptors [80]. Na_v_1.6 is also expressed in keratinocytes, which may contribute to pain sensation, and a significantly increased signal for Na_v_1.6 was found in human skin biopsies from patients with complex regional pain syndrome and post-herpetic neuralgia [81]. These data suggest a major role for Na_v_1.6 in the function and pathophysiology of small-diameter sensory nerve endings. In addition, several studies have provided strong evidence that Na_v_1.6 is the predominant sodium channel isoform expressed in microglia and contributes to the response of microglia to multiple activating signals [reviewed by 16], and microglia are known to have an important role in pathologic pain [40].

Na_v_1.6 appears not to be involved in inflammatory pain [43], and its expression was unchanged in thalamic nucleus [44], DRG neurons, and sciatic nerve [73,78] after peripheral nerve injury in rat and in human neuromas [42]. There have also been reports of Na_v_1.6 downregulation in DRG neurons after peripheral nerve injury [45,82,83,84]. Contradictory results have been obtained using the streptozotocin-induced painful diabetic neuropathy model. One group observed a significant upregulation in Na_v_1.6 mRNA and protein expression in DRG neurons [54], whereas a subsequent study found a downregulation in its protein expression in these neurons [56]. Another group reported a significantly increased Na_v_1.6 protein expression proximal to the injured site in a rat model of infraorbital nerve injury [85]. Additional research is needed to elucidate the involvement of this channel in pathologic pain.

### 3.5. Na_v_1.7

VGSC isoform Na_v_1.7 is expressed in all types of DRG neurons, sympathetic neurons, Schwann cells, and neuroendocrine cells [8,86]. Rat studies demonstrated its expression in virtually all small-diameter neurons in the DRG [39] and in most intra-epidermal nerve fibers, where it is co-localized with Na_v_1.8 [80]. Given the localization and electrophysical properties of Na_v_1.7 [36], it likely acts as a ’threshold’ channel, amplifying generator potentials and hence setting the gain in nociceptor neurons [87]. In fact, Na_v_1.7 is essential for nociception in humans, and gain-of-function mutations of its gene (SCN9A) are associated with hyperexcitability and extreme pain, while loss-of-function mutations produce insensitivity to pain [21,88,89,90].

The key role of Na_v_1.7 in nociception has been confirmed by animal research data. Na_v_1.7 mRNA and/or protein expression in DRG was upregulated in models of peripheral inflammatory pain [43,91,92] in parallel with an increase in TTX-sensitive sodium currents [43]. Na_v_1.7 expression was also upregulated by administration of nerve growth factor (NGF), an inflammatory mediator [93]. Na_v_1.7 knockdown in primary afferents in mice prevented the increased Na_v_1.7 expression in DRG neurons and development of hyperalgesia induced by the inflammatory compound complete Freund’s adjuvant [94]. Data from experiments with Na_v_1.7 nociceptor-specific knockout mice also suggested a major role for Na_v_1.7 in acute and inflammatory pain [95].

However, the role of Na_v_1.7 in neuropathic pain remains uncertain. Thus, peripheral nerve injury-induced neuropathic pain developed normally in mice lacking Na_v_1.7 [96]. In support of this finding, the injured DRG and sciatic nerve in this pain model showed reduced Na_v_1.7 protein and/or mRNA expression in rodents [45,73,82,83,84]. Na_v_1.7 protein expression was also downregulated in trigeminal ganglion of ferrets after trigeminal nerve injury [74]. However, two groups reported a significant increase in Na_v_1.7 protein expression in the DRG neurons of rats with painful diabetic neuropathy [56,97], although another study found no change in Na_v_1.7 mRNA expression [54]. A recent rat study demonstrated an accumulation of this isoform in experimental neuromas [98].

Data on patients are also inconclusive, although there is some degree of agreement with the experimental results. Na_v_1.7 expression was reduced (vs. controls) in cell bodies of injured DRG after central axotomy [51] and in the gingival tissue of patients with trigeminal neuralgia [52]. However, epidermal labeling for Na_v_1.7 was more intense in human skin biopsies from patients with complex regional pain syndrome and post-herpetic neuralgia than in non-painful skin samples [81]. Na_v_1.7 was accumulated in human painful neuromas, similar to findings in rat [42,99]. An increased axonal expression of Na_v_1.7 in painful human dental pulp was also reported [100], and a marked increase in Na_v_1.7-immunoreactive nerve fibers was found in the mucosal, sub-mucosal, and muscle layers of patients with idiopathic rectal hypersensitivity and fecal urgency [101]. Consequently, Na_v_1.7 appears to have a clear role in acute and inflammatory pain, but further studies are required to clarify its involvement in neuropathic pain.

## 4. Effects of TTX in Pain States

TTX has been extensively used in numerous laboratories to characterize the role of VGSCs in normal physiology and in disease and their involvement in the molecular mechanisms of pain. Its effects have been studied in several animal pain models, and it has also been tested for pain relief in the clinical setting. The animal and human research results are reviewed in this section.

### 4.1. Preclinical Studies

#### 4.1.1. Effects of TTX in Acute Pain

Despite the key role of the TTX-sensitive sodium channel Na_v_1.7 in nociception (see above), TTX appears to be practically unexplored in classical models of acute pain (see Table 2). In fact, most data on the effect of TTX on pain perception in non-sensitized animals derive from controls used in studies on its effect in sensitized animals. There have also been reports on the properties of TTX as a local anesthetic. However, there have been very few specific investigations on the effect of TTX in acute pain. Early studies showed that TTX applied to the cornea of rabbit was effective as a long-acting topical anesthetic [102,103]. Other authors investigated the effects of nerve blockade with TTX on sensory properties. Sciatic nerve blockade with intraneural TTX inhibited thermal and mechanical sensitivity in uninjured rats [104] but had no effect or only moderate effects on endothelin-1 (ET-1)-induced acute pain, in contrast to the effects of blockade with lidocaine [105]. Intraneural TTX significantly increased the frequency and duration of nerve blockade from tricyclic antidepressant compounds in comparison to its systemic administration [106].

Marcil and collaborators [107] tested TTX in the model of formalin test in the rat. Although the systemic administration of TTX (at the highest doses used) in rats reduced the pain score in the initial acute formalin-induced pain, the difference did not reach significance. On the other hand, TTX had a potent effect in the writhing test induced by intraperitoneal injection of acetic acid in mice [107]. In our laboratory, we showed that systemic TTX had no effect on the response to heat, mechanical, or cold stimuli in control animals, at doses effective in neuropathic pain behavior (see Table 2) [108]. We have also found that systemic TTX had no effect on mechanical nociceptive pain, whereas mexiletine (a nonspecific sodium channel blocker, like lidocaine) induced a marked antinociception, increasing the paw withdrawal latency time [109]. In another report, systemic TTX administration had no effect on the normal perception of thermal and mechanical stimuli in naïve rats [110]. Finally, in a very recent study, intrathecal TTX inhibited thermal sensitivity in intact rats, and its inhibitory potency was around 300-fold higher than that of carbamazepine, which is considered to inhibit both TTX-sensitive and TTX-resistant sodium channels [111]. These data suggest that the administration of TTX might have little impact on acute pain, although to elucidate this issue, further studies using different routes of administration are required.

#### 4.1.2. Effects of TTX in Inflammatory Pain

As in acute pain, the effects of TTX in inflammatory pain have not been well studied (see Table 2). After the initial acute pain phase induced by intraplantar formalin in rodents, this chemical algogen produces a second phase involving inflammation [112]. In contradistinction to the absence of effect in the first acute phase of formalin-induced pain (see section above), systemic TTX significantly prevented pain behavior in the inflammatory phase of the formalin test in rat [107]. In another study, contralateral and ipsilateral sciatic nerve blockade with TTX or bupivacaine (nonspecific for sodium channels) significantly attenuated mechanical and thermal hyperalgesia in response to carrageenan-induced inflammation. Systemic administration of either compound at the same dose as used in local administration (nanomolar concentration) was ineffective to prevent hyperalgesia [113]. However, a later study found that the preventive administration of systemic TTX (at micromolar concentration) slightly but significantly reduced carrageenan-induced mechanical hyperalgesia in rats [114]. Intrathecal TTX inhibited thermal hypersensitivity in a model of chronic inflammatory pain induced by complete Freund’s adjuvant (CFA). In the same study, the authors found that the relative inhibitory potency of TTX in inflamed rats was approximately 150-fold higher than that of intrathecal carbamazepine [111]. Interestingly, peri-sciatic administration of TTX has been shown to decrease carrageenan-induced edema [113], suggesting that TTX could be useful attenuating the neurogenic inflammatory response to an injury. In addition to this action of TTX in neurons, it has been proposed that sodium channel blockade with agents such as TTX or phenytoin may have anti-inflammatory effects through inhibition of the functions of some immune cells [115]. Taken together, the reports on TTX in inflammatory pain, although few in number, are promising, and given the major role in inflammatory pain attributed to TTX-sensitive VGSCs, further studies are warranted on the effects of TTX in inflammatory pain.

#### 4.1.3. Effects of TTX in Neuropathic Pain

TTX appears to have been more widely studied in models of neuropathic pain than in acute or inflammatory pain (see Table 2), probably due to the considerable evidence on the key role of TTX-sensitive VGSCs in neuropathic pain (see Section 3 and Table 1). The first study on its effects in neuropathic pain showed that pain behavior in the spinal nerve ligation model was significantly attenuated by the topical application to the DRG of TTX at low doses that did not block action potential conduction [116]; the authors reported that the most effective dose (25 nM) was also effective when applied to the epidural space but not when administered to the intraperitoneal space. A few years later, Xie *et al.*, [104] used TTX for sciatic nerve blockade in two different rat models of neuropathic pain induced by sciatic nerve injury. They showed that immediate post-injury perfusion of the injured nerve with TTX permanently prevented the development of thermal hyperalgesia, mechanical allodynia, and the spontaneous afferent activity measured with electrophysiological recordings. In contrast, when TTX was applied after a longer post-injury interval, when the neuropathy had already developed, it only transiently inhibited mechanical allodynia. These authors obtained similar results with bupivacaine. Marcil *et al.* [107] reported that systemic TTX was most effective in neuropathic pain conditions, based on significant reductions of the mechanical allodynia and thermal hyperalgesia induced by partial sciatic nerve ligation at lower doses than those required to inhibit pain behaviors induced by intraplantar formalin or intraperitoneal acetic acid. In another interesting article, Kayser *et al.* [110] reported that TTX exerted differential anti-neuropathic pain effects in sciatic nerve- *versus* infraorbital nerve-ligated rats, and the authors also contributed information on possible mechanisms underlying the anti-neuropathic pain effects of TTX. These authors showed that acute and subchronic systemic administration of TTX suppressed thermal and mechanical hyperalgesia and tactile allodynia after sciatic nerve injury, whereas acute TTX treatment had only a moderate effect after infraorbital nerve injury. They also reported that acute systemic administration of TTX prevented the increase of c-Fos expression (as neuronal activity marker) in dorsal horn of lumbar cord and some supraspinal areas in response to light mechanical stimulation of the sciatic nerve-injured hindpaw. Finally, the same study found that noradrenergic and serotoninergic systems do not appear to be involved in the anti-neuropathic pain action of TTX, whereas endogenous opioid systems may have a role [110]. In another report, the preventive topical administration of TTX to the median nerve impeded the development of mechanical hypersensitivity after its chronic constriction injury (CCI); TTX also reduced the increased astrocyte activation in the cuneate nucleus induced by this nerve injury [117].

TTX has been tested in models of chemotherapy-induced neuropathic pain with contradictory results. It was reported that the administration of systemic TTX had no effect on the expression of mechanical allodynia induced by vincristine in rats [118]. However, our group found that TTX inhibited the expression of mechanical and cold allodynia and heat hyperalgesia induced by paclitaxel in mice. We also demonstrated that TTX completely prevented the development of both kinds of allodynia but not the hyperalgesia [108]. The discrepancy between studies may be attributable to the different antitumor mechanisms of paclitaxel and vincristine. Both produce clinically significant peripheral neuropathies, whose toxic mechanisms are not fully understood but are known to vary between the anti-cancer drugs [119]. In addition, our group reported that TTX inhibited capsaicin-induced mechanical hypersensitivity in mice [109], which is considered a surrogate model of neuropathic pain [120]. Finally, perfusion of the injury-site DRG with TTX was found to significantly reduce the activation of satellite glial cells, increased NGF expression in the DRG, and activation of microglia and astrocytes in the spinal cord after peripheral nerve injury [121]. In conclusion, only one study using an adequate dose of TTX failed to inhibit neuropathic pain in rodents [118]. Therefore, these findings generally support the hypothetical therapeutic usefulness of TTX in neuropathic pain and may indicate that TTX-sensitive VGSCs play a key role in neuropathic pain states. Nevertheless, they strongly contrast with data obtained in VGSC knockout mice [76,96], although the development of compensatory mechanisms in these animals in comparison with animals treated with TTX cannot be discarded. Further research is required to elucidate this issue.

#### 4.1.4. Effects of TTX in the Electrophysiological Abnormalities Associated with Neuropathic Pain

**Figure 2:**
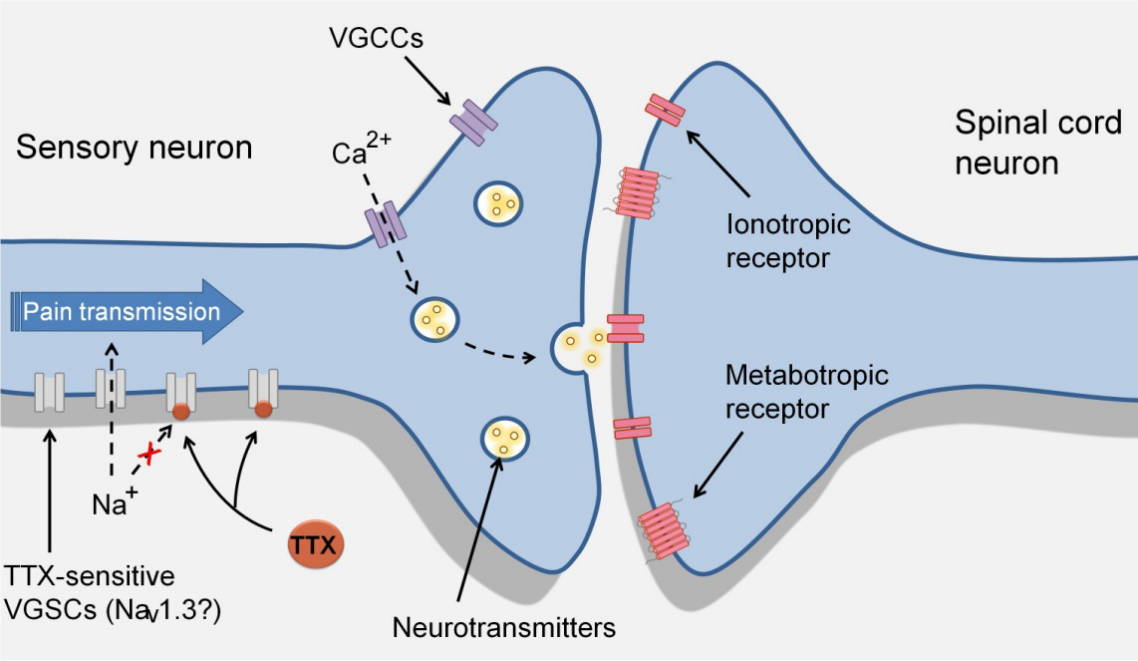
Proposed mechanism of action of TTX in sensory neurons during neuropathic pain. During neuropathy sensory neurons can produce ectopic action potentials, purportedly by the re-expression of the voltage-gated sodium channel (VGSC) Na_v_1.3. The action potential is propagated along the axon to activate voltage-gated calcium channels (VGCCs), which in turn trigger the release of neurotransmitters by the presynaptic terminal to activate their receptors in dorsal horn neurons. TTX by inactivating TTX-sensitive sodium channels such as Na_v_1.3, could prevent neuronal ectopic activity.

DRG neurons express sodium currents that contain both rapidly-inactivating TTX-sensitive components and slowly-inactivating TTX-resistant components. However, peripheral nerve injuries produce a downregulation of Na_v_1.8 and Na_v_1.9 (TTX-resistant channels) and an upregulation of Na_v_1.3 in DRG neurons [28,29,30,31,34,122], which is accompanied by the appearance of a fast repriming TTX-sensitive current [31,34]. This abnormal sodium current was identified as TTX-sensitive after experiments demonstrated that it was blocked by the application of TTX [31,33,34]. The emergence of this sodium channel current is largely imputable to the re-expression of Na_v_1.3 and likely contributes to the hyperexcitability and ectopic firing that have been observed in DRG neurons after injury (see Figure 2).

An important characteristic of neuropathic pain is spontaneous pain produced by the generation in nociceptive pathways of ectopic action potential that does not originate in peripheral terminals in response to a stimulus [40]. Topical application of TTX to rat neuromas successfully prevented ectopic discharges [123], and intravenous administration of TTX in rats inhibited post-sciatic nerve transection ectopic activity in neuromas and in DRG and dorsal horn neurons [124]. Furthermore, recordings of primary sensory neurons in excised rat DRG revealed that post-nerve injury oscillations and ectopic spiking were removed by its perfusion with TTX at doses that did not affect axonal spike propagation or block TTX-resistant sodium channels [125,126]. Therefore, inhibition by TTX of the atypical rapid sodium currents and/or abnormal ectopic discharges observed in the DRG after nerve injury may contribute to the effects of TTX against neuropathic pain. 

**Table 2:** Summary of the effects of TTX on pain studies in laboratory animals.

Type of pain	Administration of TTX	TTX doses	Effect (+, +/- or -)	Test	Reference
**Acute pain**	Sciatic nerve blockage	TTX osmotic pump	+	Thermal and mechanical sensitivity	[104]
		Intraneural (10 mM/4 µL)	+/-	Pain induced by ET-1	[105]
	Intrathecal	0.2–6 pM/20 µL	+ (2–6 pM)	Thermal hypersensitivity	[111]
	Systemic	0.3–6 µg s.c.	-	1º phase of formalin test	[107]
		0.3–6 µg s.c.	+ (3–6 µg)	Writhing test	[107]
		1–6 µg s.c.	-	Mechanical, cold and heat sensitivity	[108]
		6 µg s.c.	-	Mechanical nociceptive pain	[109]
		Acute and subchronic TTX (1–6 µg s.c.)	-	Thermal and mechanical sensitivity	[110]
**Inflammatory pain**	Sciatic nerve blockage	50 µM/0.2 mL	+	Carrageenan-induced paw inflammatory edema and mechanical and thermal hyperalgesia.	[113]
	Intrathecal	0.2–6 pmM/20 µL	+ (0.2–6 pM)	Thermal hypersensitivity induced by CFA	[111]
	Systemic	0.3–6 µg s.c.	+ (6 µg)	2° phase of formalin test	[107]
		50 µM/0.2 mL s.c.	-	Carrageenan-induced paw inflammatory edema and mechanical and thermal hyperalgesia.	[113]
		2.5 µg s.c.	+ (2.5 µg)	Carrageenan-induced mechanical hyperalgesia	[114]
**Neuropathic pain**	Sciatic nerve blockage	TTX osmotic pump	+	Thermal and mechanical hypersensitivity and spontaneous activity induced by SNI and CCI	[104]
	Topical DRG	12.5–50 nM/5 µL	+ (12.5–50 µg)	Mechanical allodynia induced by SNL	[116]
	Epidural	25 nM/5 µL	+ (25 µg)	Mechanical allodynia induced by SNL	[116]
	Topical median nerve	Gel pads with TTX	+	Mechanical hypersensitivity and the increment of astrocyte activation in the cuneate nucleus after CCI of median nerve	[117]
	Systemic	25 nM/5 µL i.p.	-	Mechanical allodynia induced by SNL	[116]
		0.3–6 µg s.c.	+ (1–6 µg)	Mechanical allodynia and thermal hyperalgesia induced by SNL	[107]
		Acute and subchronic TTX (1–6 µg s.c.)	+	Thermal and mechanical hypersensitivity and c-fos expression induced by CCI of sciatic nerve	[110]
		Acute and subchronic TTX (1–6 µg s.c.)	+/-	Thermal and mechanical hypersensitivity induced by CCI of infraorbital nerve	[110]
		8 µg i.p	-	Mechanical allodynia induced by vincristine	[118]
		1–6 µg s.c.	+	Mechanical, cold and heat hypersensitivity induced by paclitaxel	[108]
		6 µg s.c.	+	Mechanical hypersensitivity induced by intraplantar capsaicin	[109]

+: effect; +/-: moderate effects; -: no effect.

### 4.2. Clinical Studies

We know of three clinical trials on the usefulness of TTX to alleviate pain. In an open-label multicentre dose escalation study of TTX in severe cancer-related pain, 24 patients underwent 31 treatment regimens with intramuscular TTX at doses of 15–90 µg/day administered in divided doses over four days. Out of the 31 regimens, 17 yielded clinically significant reductions in pain intensity, with the pain relief persisting for up to 2 weeks. The authors concluded that 30 μg twice daily for 4 days was a regimen with an acceptable toxicity and analgesic profile [127]. In a multicenter, randomized, double-blind, placebo-controlled, parallel-design trial, the subcutaneous administration of TTX failed to deliver clinically significant pain relief in cancer patients with moderate to severe pain when only pain scores were assessed, although a strong analgesic effect was suggested by further analysis of the data [128]. At the end of the latter study, all patients were able to enroll in a multicenter open-label extension efficacy and safety trial. In this longitudinal study, 30 μg TTX was administered subcutaneously twice daily for 4 days in a heterogeneous cohort of patients with chronic cancer-related pain. The recently published results demonstrate that TTX has an acceptable tolerability, even when administered over a prolonged time period. The toxicity was typically mild and was primarily sensory and transient, with peri-oral numbness or tingling being the predominant experience [129]. Out of the 41 evaluable patients, the analgesic effect was sustained in 21 patients, whose cancer pain relief remained constant over successive treatment cycles up to and beyond 12 months, with no evidence of tolerance and an anti-nociceptive effect for an average of 3 weeks; the reason why only 50% of patients responded to TTX remains unknown. The authors suggested that further research is warranted on the use of TTX for moderate-to-severe cancer pain.

## 5. Concluding Remarks

**Figure 3:**
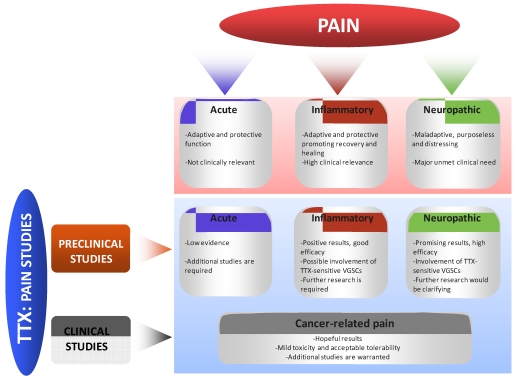
Schematic representation of the main evidences and conclusions obtained in preclinical and clinical human studies using TTX as a potential therapeutic agent for pain.

Altered expression of several TTX-sensitive sodium channels occur during pathologic pain (both inflammatory and neuropathic pain). This change in gene regulation leads to electrophysiological changes which may play a key role in the pathogenesis of the pain outcome. Therefore, the use of TTX seems reasonable as a possible pharmacological tool to block TTX-sensitive VGSCs in pathological pain conditions. Although the results obtained in preclinical inflammatory pain models are unclear at the moment, the improving effects of TTX on neuropathic pain are better documented. Consistently, TTX has been proved to be more effective against neuropathic pain than against acute nociception (in which its administration into the nervous tissue is required to show an obvious effect), probably due to the altered properties/expression of the TTX-sensitive VGSCs in pathological conditions. Importantly, TTX has been tested against cancer pain in patients, yielding promising but not conclusive results. In summary, the therapeutic use of TTX as an analgesic agent seems hopeful although further preclinical and clinical research is needed to clarify its potential use during painful conditions (see Figure 3).
